# Feasibility of Telerehabilitation Implementation as a Novel Experience in Rehabilitation Academic Centers and Affiliated Clinics in Tehran: Assessment of Rehabilitation Professionals' Attitudes

**DOI:** 10.1155/2015/468560

**Published:** 2015-11-02

**Authors:** Sara Movahedazarhouligh, Roshanak Vameghi, Nikta Hatamizadeh, Enayatollah Bakhshi, Seyed Muhammad Moosavy Khatat

**Affiliations:** ^1^Department of Rehabilitation Management, University of Social Welfare and Rehabilitation Sciences, Tehran, Iran; ^2^Pediatric Neurorehabilitation Research Center, University of Social Welfare and Rehabilitation Sciences, Tehran, Iran; ^3^Department of Rehabilitation Management, Pediatric Neurorehabilitation Research Center, University of Social Welfare and Rehabilitation Sciences, Tehran, Iran; ^4^Department of Biostatistics, University of Social Welfare and Rehabilitation Sciences, Tehran, Iran; ^5^Department of Social Welfare, University of Social Welfare and Rehabilitation Sciences, Tehran, Iran

## Abstract

*Introduction*. This study aimed to assess rehabilitation professionals' attitude toward implementation and application of telerehabilitation technology as a novel study in rehabilitation academic centers and affiliated clinics in Tehran. *Methods*. It was a descriptive cross-sectional study. To collect data, a researcher-designed questionnaire was developed. 141 rehabilitation experts participated in the study. *Results*. A majority of faculty members (78%) and clinicians (89.7%) either were in “definite agreement” or “somewhat agreed” with implementation and application of this technology, which demonstrates an overall positive attitude. *Discussion*. Based on the positive attitudes of the majority of participants toward implementation and application of this technology and their preferences in offering different telerehabilitation services, it seems that there is an appropriate and desirable acceptance and administrative culture to implement this technology among rehabilitation experts in Tehran. It is thus expected that implementation and application of this technology will be a promising experience in rehabilitation academic centers and affiliate clinics in Tehran.

## 1. Introduction

Today, new technologies have come into existence in different occupations and work areas, one of which is information and communication technology [1]. Due to rapid advances in information technology many aspects of work environments worldwide have been faced with fundamental changes. The field of healthcare has not been exempt from these effects [2] as technological aspects of patient care are constantly undergoing changes [3]. Rapid development of technology and health informatics has encouraged healthcare organizations to provide advanced services with better quality [4].

Today, healthcare organizations are faced with a new technology called telehealth. Telehealth is the use of information and communication technologies to provide remote healthcare services [5]. The World Health Organization, citing its many benefits, has stated that telehealth should be one of the main parts of healthcare strategic plans to change health systems in the 21st century [6]. Telehealth covers other specialized areas such as e-health, telemedicine, telematics, and telerehabilitation. Telerehabilitation delivers remote rehabilitation services via information and communication technologies [7].

Telerehabilitation is a relatively new area which was first defined by the National Institute of Disability and Rehabilitation in the United Sates in 1997 [8]. It encompasses a considerable range of rehabilitation services which are offered in different formats including teleassessment, teletreatment, telemonitoring, teleconsultation, telesupport, teleconferencing, teleeducation, teletherapy, telecoaching, and teleplay [9]. Telerehabilitation includes application of different technologies which can be categorized into three main modes. (1) The first mode is the store-and-forward (asynchronous) mode, in which information is recorded and stored and used when needed. As an example, in a rural community telerehabilitation site, a patient X-ray of a fracture in the knee is scanned and captured as an electronic file. This file, including accompanying medical notes, is sent electronically to the rehabilitation expert in the tertiary care telerehabilitation site. The rehabilitation professional in the tertiary care site opens the file and reviews the X-rays and notes in order to get to know about the patient's medical history and determine a general therapeutic plan. The file and accompanying notes are then returned to the rural telerehabilitation site. The patient is informed of the therapeutic plan without having to meet the physical therapist. (2) The second mode is real-time (synchronous) interactions, in which live interactive interventions take place between the service provider and receiver. As an example for the same knee fracture, here the patient and the physical therapist can arrange a video conferencing meeting in the chatroom of the rural community of the telerehabilitation site and using live interaction consultation, the therapist can instruct the patient how to do some specific exercises. (3) The third mode is the hybrid mode, which is a combination of the two mentioned modes [10]. The technologies that are used are categorized as follows: (1) textual-based technologies like e-mails, (2) audio-based (voice/sound) technologies like phones, audio recorders, and telephone answering machines, (3) vision-based technologies like video conferencing, (4) virtual reality like computer games and avatars, (5) web-based technologies like real-time chatrooms and discussion boards, (6) wireless technologies like PDAs (Personal Digital Assistants) and GPS (Global Positioning Systems), and (7) integrated systems like robots [11].

Telerehabilitation has the potential to benefit both the consumers and providers of rehabilitation services by lessening the inconvenience and/or cost of patient transfers and reducing unnecessary travel time [12]. The many benefits of this technology include elimination of distance barriers, improvement of access to healthcare services and information both for the users and for the providers in particular remote access in Iran, lessening of the inconvenience and/or cost of patient transfers and reduction of unnecessary travel time for rehabilitation professionals, and playing of an invaluable role in situations where moving a patient may be undesirable or not feasible [13]. In our country, Iran, principles of e-health have already been taken into consideration in recent years and efforts have been made to implement related systems and software [14]. It seems that factors such as rehabilitation experts' tendency to live in larger cities and, thus, lack of easy access to experts and services in smaller towns, geographical broadness, vast distribution of the population, and the existence of somewhat optimal telecommunication infrastructures in Iran can be considered as incentives for implementation of this technology in the country [15]. However, other factors can also play important roles and should not be overlooked. For example, implementation of an effective telerehabilitation system in any community requires awareness, motivation [16], and positive attitude of the people involved, in particular end-users and therapists.

Attitude plays an important role in our lives, thoughts, and our individual and social behaviors. In fact our views on various issues determine our actions and reactions and justify our motivation or reluctance to show certain behaviors [17]. Attitude toward technology is not an exception [18]. The techniques that are used for measuring attitudes, beliefs, and perceptions have gone beyond using questionnaires or having people interviewed and this is probably due to the importance of attitude studies [19]. Normally, to assess attitudes different scales such as Likert and Thurston and interview methods are used. Today, the most common way to measure attitude is grading scales among which Likert rating is the most popular [20].

In the past few years in Iran, there have been feasibility or review studies which have addressed different aspects of telemedicine and e-health including telelaparoscopy [5], telepsychiatry [21], telenursing [22], telesurgery [23], and e-health [24] but no study has ever been conducted on telerehabilitation yet. Telerehabilitation, specifically, does not even exist in the country, either in urban or in rural areas. No study has been ever conducted on telerehabilitation in the country and this research is a pioneer in this regard, both in the academic sense and in the clinical sense.

Due to the very newness of this technology in our country and considering the importance and advantages of this technology, this study aims to assess rehabilitation professionals' attitude towards implementation and application of telerehabilitation technology in rehabilitation academic centers in the city of Tehran. In the present study, attitude toward telerehabilitation implementation was defined as the respondents' views on the efficacy and the potential benefits of application of this technology in therapy and therapeutic systems.

Since rehabilitation professionals working in academic environments are expected to have more realistic attitudes towards this issue based on their more update knowledge in the rehabilitation domain and the fact that they are the most familiar ones with the current position and status of rehabilitation in the country, thus they were considered as a desirable population for conducting this study.

## 2. Method and Materials

This was a descriptive cross-sectional study conducted on faculty members of four universities and clinicians of affiliated rehabilitation centers in Tehran. A preliminary draft of an attitude questionnaire was developed by integration and cultural adaptation of different tools collected in the field of telehealth implementation. The face and content validity of the questionnaire was evaluated by a panel of rehabilitation faculty members in the University of Social Welfare and Rehabilitation Sciences using Lawshe's method and the reliability was assessed by test-retest correlation and Cronbach's alpha determinations. Finally, a researcher-designed questionnaire with favorable validity and reliability was obtained.

The designed questionnaire consisted of 51 questions scored by a Likert scale of 5, ranging from “definitely agree” with a score of 5 to “definitely disagree” with a score of 1. The higher the score, the more positive the attitude it reflected. By using 4 cut-off points for the total attainable score, 5 levels of total attitude were defined.

The questions in the questionnaire were categorized in 4 different groups, including questions that dealt with the possible positive or negative impacts of telerehabilitation implementation on (1) the experts' own work domain, (2) the work domain of colleagues, (3) the quality of therapeutic procedures and service delivery for the clients, and (4) the national health and ICT system. Overall, the questions were meant to determine what attitude the experts expressed regarding each of the suggested positive or negative outcomes or impacts of telerehabilitation implementation and application. Some examples of the questionnaire's content are stated in Table 1.

The study received an approval from the IRB and Ethical Committee of the University of Social Welfare and Rehabilitation Sciences. The study population included rehabilitation experts (faculty members and clinicians) in the University of Social Welfare and Rehabilitation Sciences and Tehran, Iran, and Shahid Beheshti Universities of Medical Sciences. Stratified sampling was used. The universities were considered as the main classes and the experts' positions as a faculty member or a clinician constituted the subclasses. The samples from each class and subclass were selected by simple random sampling. The questionnaire then was distributed manually by the researcher and participants were asked to complete the questionnaire in a few days, after which the researcher returned to collect them. Totally, 150 rehabilitation experts were recruited of which 141 participated by completing them.

## 3. Results

Table 2 shows the demographic characteristics of the participants. The results are presented in terms of two main categories: faculty members and clinicians.

Table 3 shows the distribution of the rehabilitation experts' overall attitude towards implementation and application of telerehabilitation, in terms of their groups of faculty members and clinicians. As can be seen a majority of faculty members (78%) and clinicians (89.7%) were either in “definite agreement” or “somewhat agreed” with implementation and application of this technology, which demonstrates an overall positive attitude. If “overall positive attitude” be defined as the sum of the total percentage of “definite agreement” and the total percentage of “somewhat agreement”, then according to Table 4 which shows the distribution of the rehabilitation experts' attitude in terms of their age, Table 5 which shows the distribution of the rehabilitation experts' attitude in terms of their sex, Table 6 which shows the distribution of the rehabilitation experts' attitude in terms of their educational status, Table 7 which shows the distribution of the rehabilitation experts' attitude in terms of their university of employment and Table 8 which shows the distribution of the rehabilitation experts' attitude in terms of their working experience, the highest percent of overall positive attitude was observed in participants with >30 years of age, female participants, participants with a PhD degree, participants who were employed in the Tehran University of Medical Sciences and in participants with more than 20 years of experience.

Table 9 shows the distribution of the respondents' total attitudes towards the implementation and application of telerehabilitation technology according to their specialty. As the results show speech therapy and occupational therapy were the only specialties in which some “definite agreement” overall was detected (13.3% and 2.9%, resp.). The lowest level of attitude belonged to the orthotists, 37.5% of whom had “no idea” regarding the issue and none were in “definite agreement” with it.

Table 10 shows the distribution of respondents' positive attitudes towards the implementation and use of various telerehabilitation services, in terms of their specialty. According to this table, teleconferences were the only remote service with which all respondents of all different specialties showed definite positive attitude (100% definite agreement). Other more popular telerehabilitation services included tele-experts consultation, tele-patient consultation, and tele-patient referrals. Teleevaluation seemed to be the least popular.

## 4. Discussion

Regarding the implementation and application of telerehabilitation technology, as the results show both faculty members and clinicians expressed a high percentage of positive attitude toward the implementation of telerehabilitation technology. This finding is somewhat in concordance with studies conducted by Mirhosseini et al. in the Kerman University of Medical Sciences [25] and Alizadeh in the Mazandaran University of Medical Sciences [26], both of whom demonstrated a positive attitude among medical experts to implement and apply telemedicine.

In our study clinicians surpassed the faculty members in their positive attitude toward this technology. It may be that since the majority of clinicians practice in clinical centers and are thus more familiar with the present obstacles of patient treatment they have a better understanding of the possible advantages of this technology. On the other hand, probably because faculty members in this study had higher seniority and thus more experience (see Table 2), they are more aware of the possible challenges and problems of implementation of this technology in the present situation and have a more realistic look into the matter and so tend to express a more cautious attitude.

Based on the results, with increasing age a predominantly decreasing trend of definite positive attitude has emerged. It seems that since people less than 30 years of age have more interest in and are in more contact with a variety of modern technologies they thus have a more positive view towards implementation of this modern technology. Also, our results showed that the percentage of “definite agreement” in participants with 40 to 50 years and above 50 years of age was 0%. It seems that aging has a considerable impact on the participants' cautious views, since older participants may have a better and deeper understanding about the possible challenges of implementation of this technology in our current situation, based on their higher experience. However, in a study conducted on medical students' points of view toward establishment of telemedicine in Mazandaran University of Medical Sciences, no significant correlation was found between age and attitude [26]. Evidently, in the Mazandaran study all participants were medical students whose age range was not as wide as that of the present study. Additional studies may clarify the role of age in this issue.

If “overall positive attitude” is defined as the sum of the total percentage of “definite agreement” and the total percentage of persons who responded with “somewhat agree,” our results showed that participants with a Ph.D. degree expressed more positive attitude than other degree holders. However, participants with a Ph.D. degree were the only group among whom no “definite agreement” was obtained. The findings of a study which was conducted by Alizadeh et al. showed that in Mazandaran University of Medical Sciences medical students with more years of education had more positive attitude than those with lower levels of education toward telemedicine [26].

The findings show that the University of Social Welfare and Rehabilitation Sciences was the only university in which some participants showed “definite agreement” with implementation of telerehabilitation. This may be due to the fact that this university is the only specialized university in the field of rehabilitation in Iran, with exclusive focus on welfare and rehabilitation sciences. However, if “overall positive attitude” is defined as the sum of the total percentage of “definite agreement” and the total percentage of persons who responded with “somewhat agree,” it is noteworthy that the highest percent of positive attitude belonged to the Tehran University of Medical Sciences and the lowest belonged to the Shahid Beheshti University of Medical Sciences.

As the findings show teleconferences were the most popular telerehabilitation service, followed by tele-experts consultation and tele-patient consultation, in all of which it is possible to establish a live connection between the therapist and his colleague or patient and it is also possible to benefit from both sound and image in interactions.

## 5. Conclusion

Overall, based on the results of the present study it can be anticipated that, in case of implementation of this technology in the field of rehabilitation, an overall positive trend in its acceptance and application by experts can be expected.

One of the limitations the authors faced was that to their knowledge, no study of any kind had ever been conducted before regarding telerehabilitation in Iran or even in other countries of the region. All similar studies including feasibility studies or review articles, have been conducted on different domains of telemedicine, e- helath and tele-health implementation. Thus we have been faced with lack of adequate national or regional research benefits from similar experiences in this field for further comparison and discussion of the results. It seems that further feasibility studies are required in this field in Iran and similar developing countries to enhance rehabilitation service quality by implementing telerehabilitation technology.

## Figures and Tables

**Table 1:** 

Category	Questions
Possible impacts of telerehabilitation implementation on the experts' own work domain	(i) Benefits of telerehabilitation application in enhancing professional contacts (ii) Challenges of telerehabilitation application (iii) Benefits of different modes of telerehabilitation in different rehabilitation specialties

Possible impacts of telerehabilitation implementation on their colleagues' work domain	(i) Benefits of telerehabilitation application in professional responsibilities of colleagues (ii) Ethical challenges of telerehabilitation application by colleagues (iii) Rehabilitation professionals' resistance toward telerehabilitation application

Possible impacts of telerehabilitation implementation on the treatment procedure and service delivery for the client	(i) Efficacy of telerehabilitation in different rehabilitation specialties (ii) Efficacy of telerehabilitation in different stages of therapy

Possible impacts of telerehabilitation implementation on the national supportive system	(i) Economical advantages and challenges of telerehabilitation application (ii) Reimbursement issues of telerehabilitation application (iii) Required IT infrastructures of telerehabilitation implementation

**Table 2:** Demographic characteristics of the participants.

Demographic characteristics	Participants	
	Faculty members	Clinicians
	%	%
Age		
<30	0	15.4
30–40	20.7	53.8
40–50	34.9	20.5
>50	44.4	10.3
Total	**100**	**100**
Sex		
Male	58.7	35.9
Female	41.3	64.1
Total	**100**	**100**
Educational status		
B.A.	0	30.8
M.A.	27	51.3
Ph.D./doctorate	73	17.9
Total	**100**	**100**
University of employment		
University of Social Welfare and Rehabilitation Sciences	55.6	74.4
Tehran University of Medical Sciences	4.7	13.8
Shahid Beheshti University of Medical Sciences	14.3	6.5
Iran University of Medical Sciences	25.4	5.3
Total	**100**	**100**
Working experience (years)		
<5	0	3.8
5–10	11.1	55.1
10–15	30.2	29.5
15–20	38.1	10.3
>20	20.6	1.3
Total	**100**	**100**

**Table 3:** The distribution of the rehabilitation experts' attitude in terms of their groups.

	Attitude					
	Definitely agree	Somewhat agree	Have no idea	Somewhat disagree	Definitely disagree	Total
	%	%	%	%	%	%
Groups						
Faculty members	0	77.8	22.2	0	0	100
Clinicians	3.8	85.9	10.3	0	0	100

**Table 4:** The distribution of the rehabilitation experts' attitude in terms of their age.

	Attitude					
	Definitely agree	Somewhat agree	Have no idea	Somewhat disagree	Definitely disagree	Total
	%	%	%	%	%	%
Age (years)						
<30	8.3	83.3	8.4	0	0	100
30–40	3.6	81.9	14.5	0	0	100
40–50	0	84.2	15.8	0	0	100
>50	0	80.6	19.4	0	0	100

**Table 5:** The distribution of the rehabilitation experts' attitude in terms of their sex.

	Attitude					
	Definitely agree	Somewhat agree	Have no idea	Somewhat disagree	Definitely disagree	Total
	%	%	%	%	%	
Sex						
Male	1.5	80	18.5	0	0	100
Female	2.6	84.2	15.6	0	0	100

**Table 6:** The distribution of the rehabilitation experts' attitude in terms of their educational status.

	Attitude					
	Definitely agree	Somewhat agree	Have no idea	Somewhat disagree	Definitely disagree	Total
	%	%	%	%	%	%
Educational status (degree)						
B.A.	4.2	79.2	16.6	0	0	100
M.A.	3.5	84.2	12.3	0	0	100
Ph.D./doctorate	0	88.4	11.6	0	0	100

**Table 7:** The distribution of the rehabilitation experts' attitude in terms of their university of employment.

	Attitude					
	Definitely agree	Somewhat agree	Have no idea	Somewhat disagree	Definitely disagree	Total
	%	%	%	%	%	%
University of employment						
USWR^*∗*^	3.2	80.8	16	0	0	100
TUMS^*∗∗*^	0	92.3	7.7	0	0	100
Sh.B UMS^*∗∗∗*^	0	78.6	21.4	0	0	100
IUMS^*∗∗∗∗*^	0	85	15	0	0	100

^*∗*^University Of Social Welfare and Rehabilitation Sciences.

^*∗∗*^Tehran University of Medical Sciences.

^*∗∗∗*^Shahid Beheshti University of Medical Sciences.

^*∗∗∗∗*^Iran University of Medical Sciences.

**Table 8:** The distribution of the rehabilitation experts' attitude in terms of their working experiences.

	Attitude					
	Definitely agree	Somewhat agree	Have no idea	Somewhat disagree	Definitely disagree	Total
	%	%	%	%	%	%
Working experience (years)						
<5	0	66.7	33.3	0	0	100
5–10	4	80	16	0	0	100
10–15	2.4	82.9	14.7	0	0	100
15–20	0	84.4	15.6	0	0	100
>20	0	85.7	14.3	0	0	100

**Table 9:** Distribution of the respondents' total attitude towards the implementation and application of telerehabilitation technology in terms of specialty.

	Attitude					
	Definitely agree	Somewhat agree	Have no idea	Somewhat disagree	Definitely disagree	Total
	%	%	%	%	%	
Specialty						
Physical therapist	0	91.7	8.3	0	0	100
Occupational therapist	2.9	79.5	17.6	0	0	100
Audiometer	0	90.9	9.1	0	0	100
Optometrist	0	100	0	0	0	100
Speech therapist	13.4	53.3	33.3	0	0	100
Rehabilitation consultant	0	83.3	16.7	0	0	100
Ergonomist	0	100	0	0	0	100
Orthotists	0	62.5	37.5	0	0	100
Nurse	0	100	0	0	0	100
Other specialties	2.1	91.65	6.25	0	0	100

**Table 10:** Distribution of respondents' positive attitude towards implementation of different telerehabilitation services in terms of specialty.

Telerehabilitation service	Specialty									
	Physical therapist (%)	Occupational therapist (%)	Audiologist (%)	Optometrist (%)	Speech therapist (%)	Rehabilitation consultant (%)	Ergonomist (%)	Orthotists (%)	Nurse (%)	Other specialties (%)
Tele-expert consultations	100	24.1	90.1	100	100	100	100	100	100	100
Teleconferences	100	100	100	100	100	100	100	100	100	100
Tele-follow-ups	75	73.2	72.7	62	60	83.4	100	25	80	79.1
Tele-patient referrals	95.7	91.1	7.8	100	93.4	100	100	87.5	100	100
Tele-patient assessment	58.3	52.9	45.5	50	46.6	66.7	100	12.5	60	80
Telemonitoring	66.7	64.7	63.6	62.5	16.6	66.7	100	12.5	80	80
Teleevaluation	45.8	60	45.5	50	40.7	33.3	50	12.5	60	47.9
Tele-patient consultation	100	91.2	100	100	100	100	100	100	100	100
